# GDF15 Drives Glioblastoma Radioresistance by Inhibiting Ferroptosis and Remodeling the Immune Microenvironment

**DOI:** 10.7150/ijbs.115721

**Published:** 2025-10-20

**Authors:** Wenqing Feng, Yantan Liu, Qinghua Zhang, Shushu Hu, Dehuang Xie, Peixin Tan, Yuan Lei, Chen Chen, Chen Ren, Shasha Du

**Affiliations:** Department of Radiation Oncology, Guangdong Provincial People's Hospital (Guangdong Academy of Medical Sciences), Southern Medical University, Guangzhou, 510080, Guangdong, China.

## Abstract

Radiotherapy is a primary treatment for glioblastoma (GBM), yet its effectiveness is limited by frequent recurrence due to radioresistance. Our previous studies have illustrated that GDF15 is highly expressed in radioresistant GBM cells and correlates strongly with recurrent GBM tissue. However, its role in radioresistance remained unclear. Here, we demonstrate that GDF15 promotes radioresistance by suppressing ferroptosis and altering the immune microenvironment. Mechanistically, GDF15 alleviates radiation-induced ferroptosis by stabilizing NRF2 protein through reduced ubiquitin-mediated degradation. Additionally, after radiation, GDF15 promotes M2 type macrophage infiltration, fostering an immunosuppressive microenvironment that further supports radioresistance. These findings emphasize GDF15 as a key mediator of GBM radioresistance and a potential therapeutic target to improve radiotherapy outcomes.

## Introduction

Glioblastoma (GBM) is among the most aggressive and lethal brain tumors, with poor survival despite surgery, radiotherapy (RT), and chemotherapy 1. Although initial treatment can shrink tumors, most patients relapse within a few months 1-3. Recurrence often arises at the margins of the radiation field, indicating survival of radioresistant clones 4. Elucidating the mechanisms of this resistance is essential to improve outcomes and reduce recurrence.

Radiation resistance in tumors is a multi-factorial process driven by both intrinsic cellular mechanisms and the tumor microenvironment 5, 6. At the cellular level, cancer cells evade radiation-induced death through diverse survival pathways 7-10. Ferroptosis, an iron-dependent form of cell death, has been increasingly associated with therapy resistance 11-14. By suppressing ferroptosis, tumor cells withstand radiation-induced damage 10, 15-17. The tumor microenvironment further contributes to radioresistance 18, 19. Its heterogeneity, particularly hypoxic regions, reduces radiation efficacy. Immunosuppressive cells such as regulatory T cells (Tregs) and myeloid-derived suppressor cells (MDSCs) infiltrate the tumor, impairing immune surveillance and supporting tumor persistence 20-23. Moreover, cytokines and growth factors from tumor and stromal cells modulate radiation responses, creating an immune-suppressive niche that fosters tumor survival and recurrence 19, 24, 25.

Among these factors, growth differentiation factor 15 (GDF15), a stress-responsive cytokine, is upregulated in multiple cancers, including GBM, and correlates with poor prognosis and therapy resistance 26-29. Accumulating evidence indicates that GDF15 expression is elevated in GBM and correlates with tumor progression and an immunosuppressive microenvironment 30-32. In radiotherapy, GDF15 is markedly induced by radiation in several cancers, suggesting its role in acquired radioresistance 33-39. For instance, studies in head and neck squamous cell carcinoma and lung cancer report that GDF15 overexpression promotes radioresistance by enhancing DNA repair and inhibiting apoptosis 35, 39. However, the precise mechanisms by which GDF15 mediates radioresistance, especially in GBM, remain unclear. Given GDF15's involvement in tumor survival and immune modulation, clarifying its function in GBM radioresistance is critical. This study explores the role and mechanism of GDF15 in GBM radioresistance, aiming to identify novel strategies to improve treatment and prevent recurrence.

## Materials and Methods

### Bioinformatics Analysis

Bioinformatics analysis was performed using publicly available datasets from Chinese Glioma Genome Atlas (CGGA, http://www.cgga.org.cn) and Gene Expression Omnibus databases (GEO). Data processing and analysis were performed in RStudio software (R version 4.2.3).

### Patient Specimens

Archived GBM specimens were retrospectively collected from Guangdong Provincial People's Hospital with approval from the institutional ethical committee. Formalin-fixed, paraffin-embedded sections associated with clinical data, including demographics and treatment history, were collected. Samples were used for histological and immunohistochemical analyses to assess tumor molecular characteristics. All patient information was anonymized, and procedures complied with ethical standards and regulatory requirements.

### Immunohistochemistry

Paraffin-embedded tissue sections were deparaffinized, rehydrated through sequential ethanol washes, and subjected to antigen retrieval in citrate buffer (pH 6.0, Servicebio, G1201) at 100°C for 30 minutes. Slides were incubated with primary antibodies at 4°C for 16 hours, followed by HRP-conjugated secondary antibodies. Immunoreactivity was visualized using DAB and counterstained with hematoxylin. Images were captured using a light microscope (Olympus, Japan). Detailed antibody information is provided in Supplementary Table 1.

### Cell Culture

Cells are maintained at 37°C in a humidified incubator with (Thermo Fisher Scientific, USA) 5% CO_2_. Human glioblastoma cell lines (U251, LN229, M059K, and M059J), murine glioma cells GL261, and human embryonic kidney cells (HEK293T) were propagated in DMEM (Procell, China) supplemented with 10% fetal bovine serum (FBS) (ExCell Bio, China). THP-1 cells were grown in RPMI-1640 medium (Procell, China) with 10% FBS (ExCell Bio, China, Cat#FSP500) and 1% penicillin-streptomycin (Beyotime, Cat#C0222). Cells were passaged every 2-3 days when reaching 80-90% confluence. Glioblastoma cell lines M059K and M059J were obtained from American Type Culture Collection (ATCC, Cat# CRL-2366 and CRL-2365).

### Gene Overexpression and Knockdown Experiments

Lentiviruses encoding GDF15 and control shRNAs (shGDF15, shNC) were purchased from Tsingke Biotechnology (Beijing, China). Transduction was performed following the manufacturer's instructions. Knockdown and overexpression efficiencies were confirmed by Western blot.

### Western Blot

Cell lysates were prepared using RIPA buffer (Beyotime, Cat#P0013B) containing a complete protease inhibitor cocktail (Roche, Cat# 04693132001). Equal amounts of protein were separated by SDS-PAGE and transferred to PVDF membranes (Millipore, USA). Membranes were blocked with 5% non-fat milk and incubated overnight with primary antibodies, followed by secondary antibody incubation. Protein bands were visualized using the ECL detection system (FDbio, FD8030). Detailed antibody information is provided in Supplementary Table 1.

### Flow Cytometric Analysis

Cells were plated in triplicate in 6-well plates and exposed to 8 Gy irradiation. Forty-eight hours post-radiation, cells were incubated with 5 μM BODIPY 581/591 C11 dye (Thermo Fisher, Cat# D3861) in serum-free medium for 30 minutes at 37°C in a humidified incubator with 5% CO_2_. After rinsing with cold PBS, cells were resuspended as and used to quantify lipid peroxidation level using flow cytometry (BD Bioscience, USA). Single-cell suspensions from intracranial GL261 tumors in C57BL/6 mice were prepared via mechanical and enzymatic dissociation. After red blood cell lysis, cells were stained with a fixable viability dye and fluorochrome-conjugated antibodies against CD45, CD11b, F4/80, CD86, and CD206 (Supplementary Table 1). Flow cytometry was performed on a BD LSRFortessa. Live CD45⁺ cells were gated, with macrophages identified as CD11b⁺F4/80⁺ and M1/M2 subsets defined by CD86/CD206 expression. Data were analyzed using FlowJo v10.8.

### GSH and MDA Assays

Intracellular glutathione (GSH) levels were quantified using a GSH detection kit (Solarbio, BC1175), and lipid peroxidation was assessed by measuring malondialdehyde (MDA) using an MDA assay kit (Solarbio, BC2005). All procedures were conducted according to the manufacturer's instructions.

### Transmission Electron Imaging

Cells were fixed in 2.5% glutaraldehyde and post-fixed with 1% osmium tetroxide. After dehydration through evaluated alcohols, samples were embedded in epoxy resin. Ultrathin slices (60-80 nm) were prepared using an ultramicrotome, stained with uranyl acetate and lead citrate, and examined using a transmission electron microscope (Hitachi, model: HT7800).

### Mouse Models

Female BALB/c-Nude and C57BL/6 mice (4-6 weeks old, n=5) were intracranially injected with 5x10^5^ luciferase-labeled cells to establish orthotopic glioblastoma xenografts. Cells were delivered to the right striatum of mice at coordinates relative to the bregma: 2.0 mm lateral, 0.5 mm anterior, and 3.0 mm depth. Mice were randomly assigned to experimental groups 1-2 weeks post-injection. Tumor growth size was monitored using an *in vivo* imaging system (IVIS Spectrum, PerkinElmer; Living Image® Software v4.5). Imaging data were analyzed to evaluate tumor progression and treatment effects. Group sizes, specified in figure legends, were selected based on previous studies and our laboratory experience to ensure adequate statistical power, consistent with standard practice in the field 40.

### Transient Transfection

HEK293T cells were transfected at 50-60% confluence using the PEI transfection method. Plasmid DNA (purchased from MiaoLing Plasmid Platform, Wuhan, China) was mixed with PEI (1mg/ml, Polysciences, 49553-93-7) at an appropriate ratio and added to the cells. After 48 hours, cells were collected for protein extraction and coimmunoprecipitation assays to assess protein-protein interactions.

### Coimmunoprecipitation

Cells were lysed in IP buffer (Abbkine,#BMP2020) supplemented with protease inhibitors (1:99, Fubio, FD1001). Protein extracts were incubated with primary antibody at 4°C for 12 hours, followed by capture with protein A/G beads (MCE, HY-K0202) for 2 hours. After four washes with ice-cold PBS, bound proteins were eluted in 1x SDS buffer (Fubio, FD006) and analyzed by Western blotting. Protein bands were detected using chemiluminescence. Antibody details are provided in Supplementary Table 1.

### qRT-PCR

Total RNA was extracted using an RNA extraction kit (EZB, China,#B0004DP) and reverse-transcribed into cDNA using a kit (AG, China). qRT-PCR was performed using SYBR Green PCR master mix (AG, China) and gene-specific primers on a real-time PCR system. The cycling conditions were 95°C for 10 minutes, followed by 40 cycles of 95°C for 15 seconds and 60°C for 1 minute. Relative gene expression was calculated using the comparative Ct method (2^-ΔΔCt^) with GAPDH as the endogenous control. Primer sequences are listed in Supplementary Table 2.

### Cell Viability and Colony-forming Assays

Cells were seeded in tissue culture-treated 96-well microplates at 1000 cells/well and irradiated with 8 Gy (Clinac 23EX Linear Accelerator, Varian, USA). Cell viability was quantitatively assessed at 24, 48, and 72 hours post-irradiation using the CCK-8 kit (Dojindo, Japan, #CK04-01). Briefly, 10 µL of CCK-8 solution was added per well, incubated for 2 hours under standard culture conditions, and the absorbance was read at 450 nm. For clonogenic survival assays, 500 cells/well were seeded in tissue culture-treated 6-well plates and exposed to 0-6 Gy the following day. After 10-14 days under standard conditions, colonies were fixed with ice-cold methanol, stained with 0.1% methyl violet solution, and colonies containing ≥50 cells were counted. Colony formation efficiency was calculated as previously described 41.

### Immunofluorescence Assay (IF)

Cells were collected 24 hours after 8 Gy irradiation, fixed with 4% paraformaldehyde, and permeabilized with 0.3% Triton X-100 for 15 minutes. After blocking with 4% BSA at room temperature for 1 hour, cells were incubated with primary antibodies for 12 hours at 4°C, followed by secondary antibodies for 2 hours at 20-25°C. Nuclei were stained with DAPI, and images were captured using a confocal laser microscope (Leica, model: TCS SP8; software: LAS X, v3.5.7). Antibody details are provided in Supplementary Table 1.

### Data Analysis

Data are presented as mean ± SEM. Normality and homogeneity of variance were assessed using the Shapiro-Wilk and Levene's tests, respectively. Two-group comparisons were analyzed using two-tailed, unpaired Student's t-tests, while multi-group comparisons were evaluated by one-way or two-way ANOVA. Kaplan-Meier survival analysis was performed to evaluate the association between GDF15 expression and survival in GBM mice. Statistical significance was defined as *p < 0.05, **p < 0.01, ***p < 0.001, and ****p < 0.0001.

## Results

### GDF15 Expression Is Increased in Recurrent GBM and Correlates with Radioresistance

**Figure 1:**
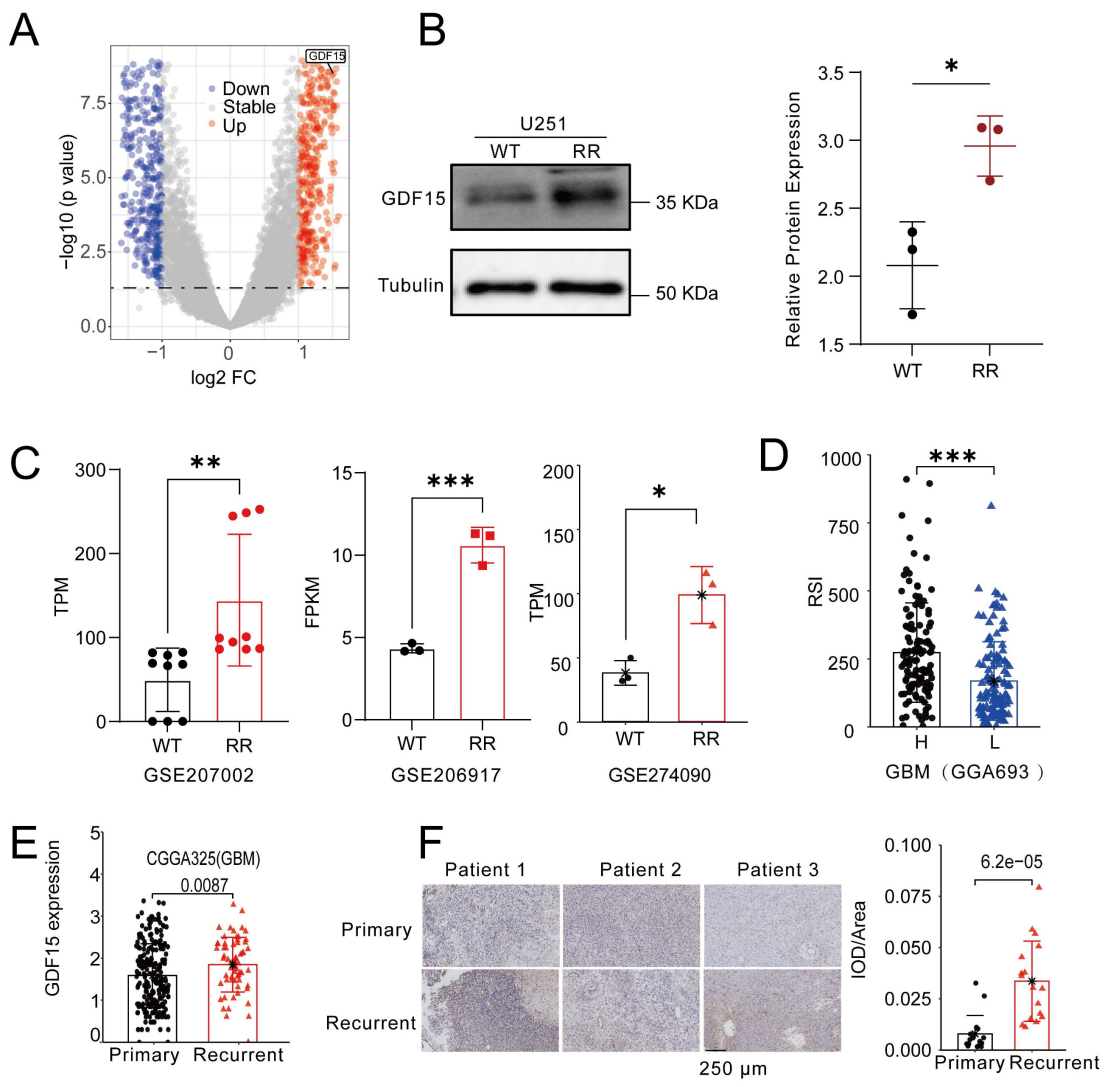
** GDF15 is upregulated in recurrent GBM and positively correlated with radioresistance. (A)** Volcano showing differential gene expression between M059K and M059J. Genes with significantly upregulated expression in M059K are shown in red, while those with significantly downregulated expression are shown in blue. Genes with no significant change in expression are represented in gray. **(B)** Western blot analysis revealing higher protein levels of GDF15 in the radioresistant U251 cell line. The intensity of the bands was quantified using ImageJ, and the protein expression was normalized to Tubulin. Statistical significance is indicated by *p < 0.05. **(C)** Transcriptome analysis from public datasets showing upregulated expression of GDF15 in the radioresistant glioma cell line compared to the parental cell line. Data were obtained from GSE207002, GSE206917, GSE274090. **(D)** Boxplot showing the Radiation Sensitivity Index (RSI) for high versus low expression groups of GDF15 in GBM patients. The RSI was calculated based on ten gene sets, with higher RSI values indicating lower radiation sensitivity. **(E)** CGGA325 datasets showing GDF15 expression in primary and recurrent GBM tissues (N=291, unit=log10Counts). **(F)** Representative IHC images of GDF15 expression in primary and recurrent GBM tissues (N=3). Scale bar: 250 μm. IOD/Area quantification was performed by calculating the total optical density (IOD) within the defined ROI divided by the area of the ROI (Area). Higher IOD values indicate stronger protein expression. Data were represented as mean ± SEM. ns, not signifcant; *, p<0.05; **, p<0.01; ***, p<0.001.

To identify key genes linked to radioresistance in glioblastoma, we compared transcriptomes of the M059K (radioresistant) and M059J (radiosensitive) cell lines. Volcano plot analysis revealed marked upregulation of GDF15 in M059K cells (Fig. 1A). To validate this finding, we generated a radiation-resistant U251 cell line (Supplementary Fig.1A-F), and Western blotting confirmed significantly increased GDF15 protein levels compared to control cells (Fig. 1B). Further analysis of GBM cell lines known for radiation resistance, including U87 and U251, revealed significant GDF15 upregulation (Fig. 1C; GSE207002, GSE206917, GSE274090). Using the radiosensitivity index (RSI) 42, which inversely correlates with radiosensitivity, we found that GBM samples with low GDF15 expression exhibited lower RSI values, indicating higher radiosensitivity (Fig. 1D; CGGA693). Since radioresistance drives GBM recurrence, we examined GDF15 expression in public datasets. Analysis of the CGGA cohort (CGGA325) revealed higher GDF15 expression in recurrent versus primary GBM (Fig. 1E). This pattern was also confirmed in clinical samples, with immunohistochemistry revealing elevated GDF15 in recurrent GBM tissues compared to their primary counterparts (Fig. 1F).

### GDF15 Contributes to Radioresistance in GBM

**Figure 2:**
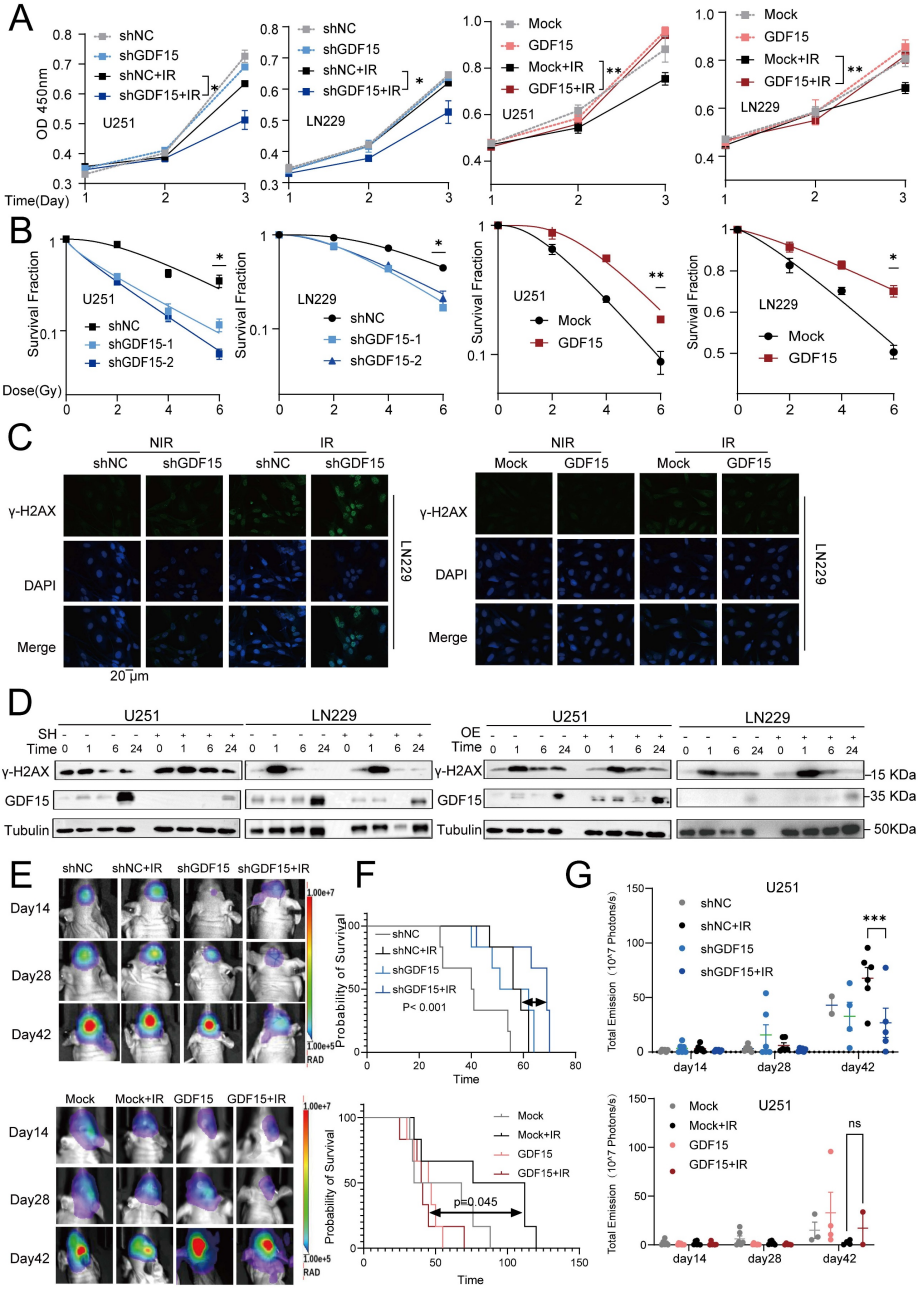
** GDF15 mediates radio-resistance in GBM.** (A) CCK-8 assay results showed that knockdown of GDF15 in GBM cells (U251 and LN229) significantly reduces cell viability following 8 Gy irradiation, while the overexpression group enhances cell viability under the same conditions. (B) Colony formation assay was conducted to assess the survival rates of GBM cells following ionizing radiation. Cells were irradiated with X-rays at doses of 0, 2, 4 and 6 Gy after overexpression or knockdown of GDF15. Survival curves were generated by plotting radiation dose on an arithmetic x-axis against surviving fraction on a logarithmic y-axis. The data were analyzed and plotted using a linear-quadratic model. (C) Immunofluorescence analysis was conducted to quantify γ-H2AX foci formation in LN229 cells following GDF15 modulation under 8 Gy X-ray irradiation at 24 hours. Results demonstrated that GDF15 overexpression significantly attenuated the formation of γ-H2AX foci post-irradiation, whereas knockdown enhanced its accumulation compared to controls (scale bar = 20 μm). Quantitative analysis illustrates the average number of foci per cell across 4 randomly selected images under each condition. (D) Western blotting showed that knockdown GDF15 delayed the removal of γ-H2AX foci in GBM cells post radiation, while overexpression group promoted the disappearance. (E) Representative *in vivo* bioluminescence imaging (BLI) at weeks 2, 4, and 6 post-implantation, showing tumor progression in different group: shNC (Control), shGDF15 (GDF15 knockdown), shNC + IR (Control+10 G y whole brain irradiation), shGDF15+ IR (GDF15 knockdown+10 Gy), Mock (Vector), Mock + IR (Vector +10 Gy), GDF15 (GDF15 overexpression), and GD15 + IR (GDF15 overexpression+10 Gy). Each group included 6 mice (n = 6 per group). (F) Kaplan-Meier survival curves of mice implanted with U251 cells under the indicated treatments. Each group included 6 mice (n = 6 per group). Survival differences were assessed by log-rank test. (G) Quantification of BLI signal intensity at weeks 2, 4, and 6 post-implantation, highlighting differential tumor growth rates among groups. Each group included 6 mice (n = 6 per group). Data are presented as mean ± SEM. ns, not significant; *p < 0.05; **p < 0.01; ***p < 0.001.

Alterations in GDF15 expression in the irradiated GBM cells were analyzed via Western blot. GDF15 protein levels in U251 and LN229 cells increased significantly after exposure to 2, 4,6 and 8 Gy compared to nonirradiated controls (Supplementary Fig. 1 G). In a time-course experiment with 4 Gy irradiation, GDF15 levels peaked at 24 hours post-irradiation in U251 cells (Supplementary Fig.1 H). To investigate GDF15's role in radioresistance, we constructed stable GDF15 knockdown and overexpressing LN229 and U251 cell lines. Following radiation, CCK8 assays demonstrated reduced cell survival in sh-GDF15 cells, while GDF15 overexpression significantly enhanced cell proliferation (Fig. 2A). Clonogenic survival assays confirmed that sh-GDF15 cells exhibited significantly lower survival fractions across radiation doses, while overexpressing cells exhibited higher survival compared to controls (Fig. 2B). Since DNA repair capacity influences radiosensitivity, we assessed double-strand break (DSB) repair using γ-H2AX. Immunofluorescence at 24 hours post-radiation revealed more γ-H2AX foci in sh-GDF15 LN229 cells, whereas GDF15 overexpression reduced foci formation (Fig. 2C, Supplementary Fig. 2A-B). Western blot analysis revealed that γ-H2AX levels declined rapidly in GDF15-knockdown GBM cells, whereas the reduction was delayed in GDF15-overexpressing cells following irradiation (Fig. 2D, Supplementary Fig. 2C-F). *In vivo*, we established orthotopic glioblastoma models using U251, LN229 cells in nude mice and GL261 cells in C57 mice. Consistent with *in vitro* results, tumors in the shGDF15 group were significantly smaller, and mice exhibited prolonged survival following radiation compared to shNC. Conversely, GDF15 overexpression tended to reduce survival (Fig. 2E-G, Supplementary Fig. 2 G-O).

### GDF15 Inhibits Ferroptosis to Reduce GBM Radiosensitivity

**Figure 3:**
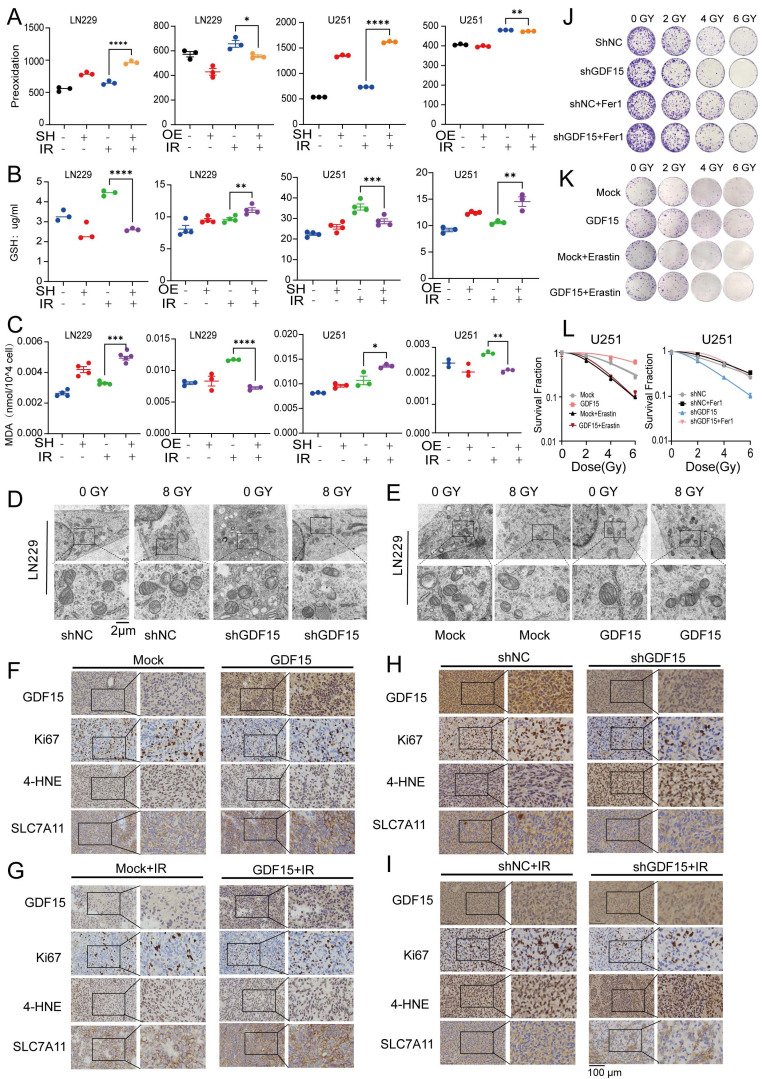
** GDF15 decreases GBM radiosensitivity by inhibiting ferroptosis.** (A) Detection of lipid peroxidation after overexpression or knockdown of GDF15 treated with 8 Gy irradiation in U251/LN229 cells. (B) Detection of GSH levels after overexpression or knockdown of GDF15 treated with 8 Gy irradiation in U251/LN229 cells. (C) Detection of MDA levels after overexpression or knockdown of GDF15 treated with 8 Gy irradiation in U251/LN229 cells. (D-E) Transmission Electron Microscopy (TEM) images of L229 cell after overexpression or knockdown of GDF15 treated with 8 Gy. Scale bar: 2 μm. (F-I) Representative images of IHC staining (GDF15, Ki67, 4-HNE, and SLC7A11) of U251 xenograft tumors with the indicated treatments. Scale bars: 100 µm. (J-K) Representative images of colony formation assay in U251 cells with GDF15 over-expression or knockdown, treated with either ferroptosis inhibitor fer1 or inducer erastin after radiation at doses of 0, 2, 4 and 6 Gy. (L) Quantification of colony formation efficiency in each treatment group. (*p < 0.05). Data were represented as mean ± SEM. ns, not signifcant; *, p<0.05; **, p<0.01; ***, p<0.001.

To explore the mechanisms of GDF15-mediated radioresistance, we performed transcriptomic analysis of LN229 cells with GDF15 knockdown after radiation. Pathway enrichment analysis revealed significant upregulation of iron related transport pathways in these cells (Supplementary Fig.3.1). Previous studies have demonstrated that ferroptosis represents a critical form of cancer cell death induced by irradiation 43, 44. To verify this in GBM cells, we first assessed the levels of malondialdehyde (MDA), GSH, and lipid peroxidation following irradiation (Supplementary Fig. 3A-D). Our results indicated that radiation promoted ferroptosis in GBM cells. We next investigated the role of GDF15 in regulating ferroptosis in GBM cells following irradiation. After irradiation, shGDF15 cells exhibited increased lipid peroxidation compared with controls, as assessed by C11-BODIPY staining, whereas GDF15-overexpressing cells showed reduced levels (Fig. 3A). Correspondingly, GSH levels decreased in shGDF15 cells and increased in GDF15-overexpressing cells (Fig. 3B). MDA measurements further confirmed these differences among the groups (Fig. 3C). Transmission electron microscopy revealed typical ferroptotic features in shGDF15 cells, including smaller mitochondria with dense membranes, reduced or absent cristae, ruptured outer membranes, cell membrane breakage, and cytoplasmic swelling; these features were attenuated in GDF15-overexpressing cells (Fig. 3D-E). *In vivo*, immunohistochemical analysis of GBM tissues from nude mice showed that tumors with high GDF15 expression had increased Ki67 and SLC7A11, but decreased 4-HNE, a representative lipid peroxidation product (Fig. 3F-I). Quantitative analyses of these markers are shown in Supplementary Fig. 3 E-P. Finally, colony formation assays using ferroptosis inhibitors and inducers confirmed the role of ferroptosis in GDF15-mediated radioresistance. The ferroptosis inhibitor ferrostatin-1 (Fer-1) reversed the radiosensitization in sh-GDF15 cells, whereas the ferroptosis inducer erastin rescued the radioresistant phenotype induced by GDF15 overexpression (Fig. 3J-L).

### GDF15 Stabilizes NRF2 to Mitigate Radiation-induced Ferroptosis in GBM

**Figure 4:**
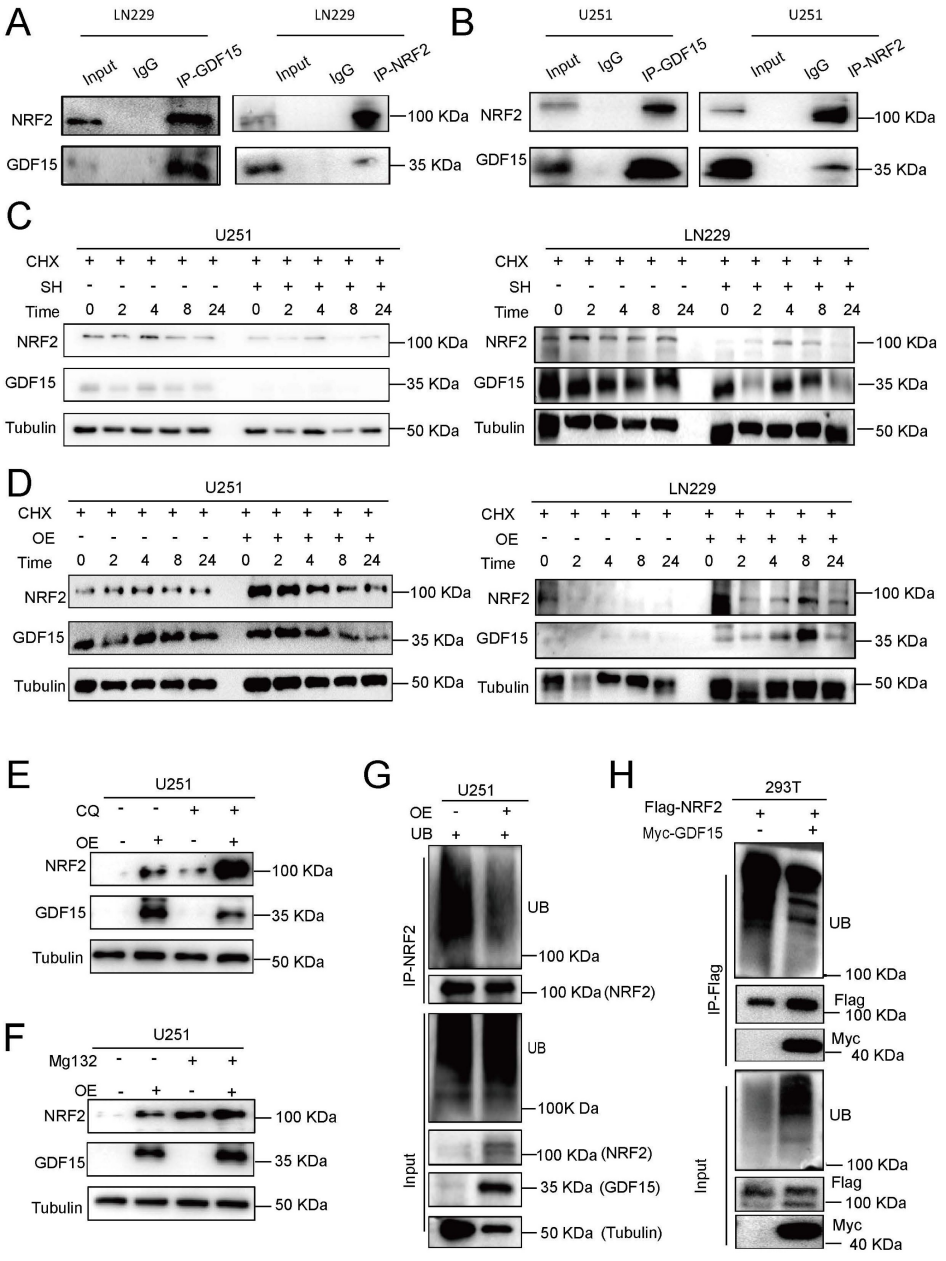
** GDF15 reduces radiation-induced ferroptosis in GBM by stabilizing NRF2 expression.** (A-B) Co-immunoprecipitation (Co-IP) analysis demonstrates interaction between GDF15 and NRF2 proteins in LN229 and U251 cells. The presence of GDF15-NRF2 protein complexes was confirmed by Western blot. (C-D) Cycloheximide (CHX) chase assay showing the degradation kinetics of NRF2 in the presence or absence of GDF15. Cells were treated with CHX (50 μg/mL) to inhibit de novo protein synthesis, and NRF2 levels were analyzed by Western blot at 0, 2, 4, 8, and 24 hours post-treatment. (E-F) NRF2 detection in U251 cells transfected with GDF15 overexpression; each group was treated with or without proteasome inhibitor MG132 or lysosomal inhibitor CQ. (G) GDF15 was stably overexpressed in U251 cells. NRF2-IP antibody was used to immunoprecipitate endogenous NRF2 protein, and ubiquitinated NRF2 in the immunocomplexes was detected with Ub antibody via WB assay. (H) HEK293T cells were co-transfected with Flag-NRF2 and Myc-GDF15 plasmids, Flag antibody was used to immunoprecipitate exogenous NRF2 protein and WB was used to validated its ubiquitination levels.

NRF2 is a key regulator of ferroptosis 45-50. To explore the relationship between GDF15 and NRF2, we first measured NRF2 mRNA levels in response to GDF15 modulation. qRT-PCR revealed that GDF15 knockdown or overexpression did not significantly alter NRF2 mRNA levels (Supplementary Fig. 4 A-B), whereas Western blot analysis showed a positive correlation between GDF15 and NRF2 protein expression both in LN229 and U251 cells under irradiation (Supplementary Fig. 4 C-H). Endogenous coimmunoprecipitation in LN229 and U251 cells confirmed a physical interaction between GDF15 and NRF2 (Fig. 4A-B). To assess post-translational regulation, cycloheximide (CHX) chase assay was performed. CHX is a well-established inhibitor of protein translation, which allows assessment of protein stability independent of new protein synthesis. We treated GBM cells with CHX to block translation and monitored NRF2 protein levels over time under GDF15 knockdown and overexpression conditions. Our results show that NRF2 decays faster in GDF15-knockdown cells and is more stable in GDF15-overexpressing cells, indicating that GDF15 prolongs the half-life of NRF2.

As shown in Figure 4C, GDF15 knockdown accelerated NRF2 degradation, shortening its half-life, while GDF15 overexpression prolonged NRF2 stability (Fig. 4D). To investigate the degradation pathway, GBM cells were treated with the proteasome inhibitor MG132 or the lysosomal inhibitor chloroquine (CQ). MG132 rescued NRF2 upregulation in GDF15-overexpressing cells, whereas CQ had no effect, indicating proteasomal regulation (Fig. 4E-F, Supplementary Fig. 4I-M). Endogenous and exogenous ubiquitination assays further revealed that GDF15 reduced NRF2 ubiquitination in LN229, U251, and 293T cells (Fig. 4G-H, Supplementary Fig. 4O), demonstrating that GDF15 stabilizes NRF2 by inhibiting its proteasomal degradation.

### GDF15 Promotes Radioresistance by Shaping Tumor Microenvironment

**Figure 5:**
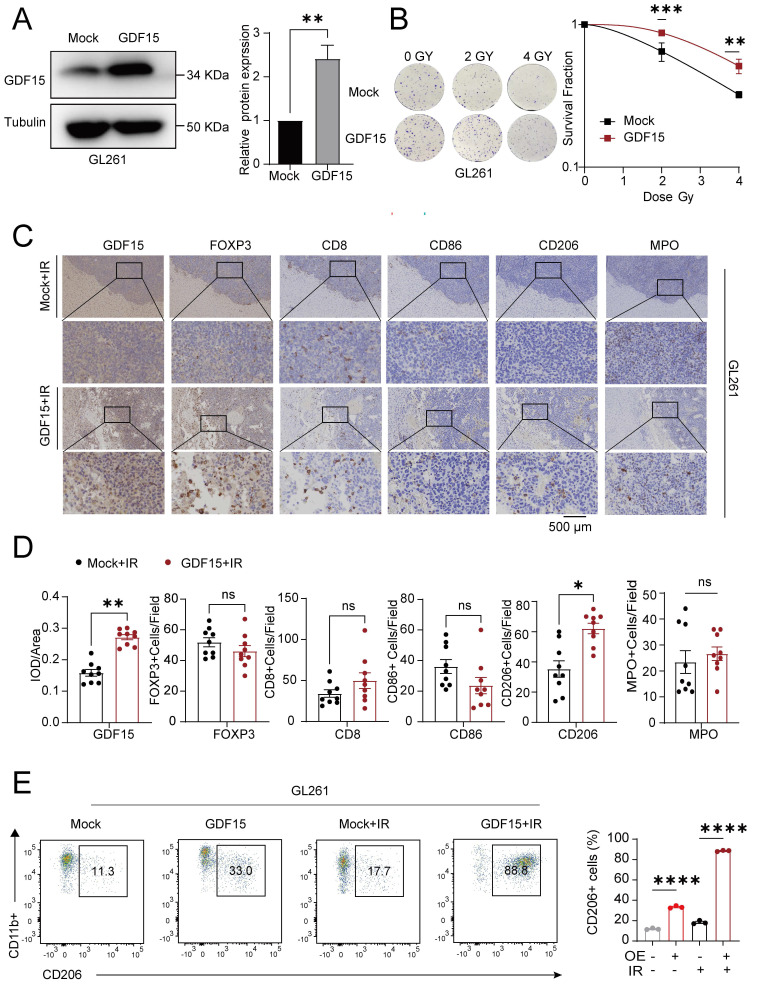
** GDF15 in glioblastoma mediates tumor-associated immunosuppression during radiotherapy.** (A) Representative Western blot showing stable GDF15 overexpression in GL261 cells. (B) Colony formation assays were performed to assess the survival of GL261 cells overexpressing GDF15 after ionizing radiation. Cells were exposed to X-rays at doses of 0, 2 and 4Gy. Survival curves were generated by plotting radiation dose on a linear x-axis against surviving fraction on a logarithmic y-axis, and data were fitted using the linear- quadratic model. (C) IHC analysis of tumor tissues from C57BL/6 mice overexpressing GDF15 after radiation therapy. Each group included exactly three mice (n = 3 per group). GDF15-overexpressing tumors showed significantly increased M2 macrophage infiltration (two-tailed t-test; scale bar, 500 μm). (D) IHC quantification of immune cells in irradiated GBM tissues from immunocompetent mice with over-expressing GDF15. Each group included three mice, with three random sections per mouse quantified. (E) Representative flow cytometry plots showing M2 macrophages ratios and quantitative analysis in aortas extracted from C57BL/6 mice after varied treatments. Each group included exactly three mice (n = 3 per group). Data were represented as mean ± SEM. ns, not signifcant; *, p<0.05; **, p<0.01; ***, p<0.001.

**Figure 6:**
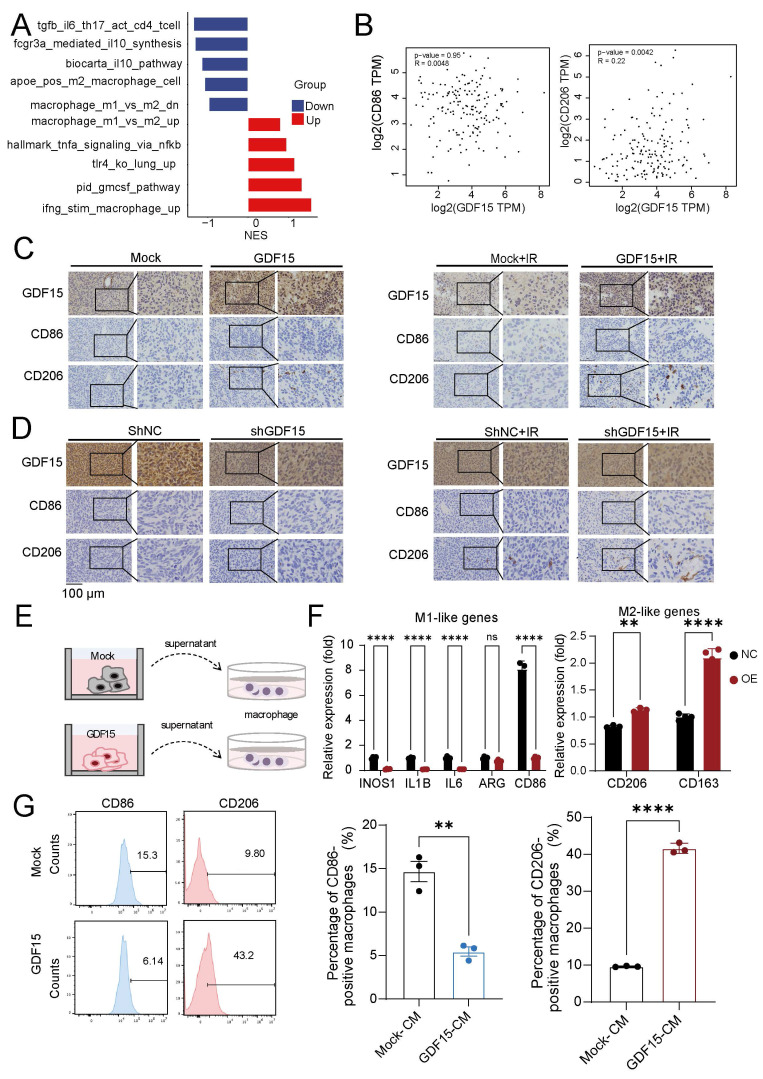
** GDF15 mediates radiotherapy resistance by promoting M2 macrophage-driven immunosuppression.** (A) Gene set enrichment analysis (GSEA) demonstrating significant upregulation of M1 macrophage polarization-related pathways and downregulation of M2 macrophage polarization-related pathways upon GDF15 gene knockdown in LN229 cell. Normalized enrichment score (NES). (B) The correlation between the expression levels of GDF15 and CD86, CD206 was assessed using data from the public database (GBM-TCGA, GEPIA2). (C-D) Representative images of IHC staining for GDF15, Ki67, CD206, CD86 in sections of U251 cell xenografts from each group. Scale bars: 100 μm. (E) Schematic model illustrating the co-culture of tumor cell supernatant with macrophages. (F) qRT‒PCR analysis of the mRNA expression levels of the M1 polarization marker and the M2 polarization marker from THP1 treated with different tumor cell supernatants. (G) Flow cytometry analysis revealed that CD206 expression was significantly increased in the GDF15 overexpression group compared with the control group while CD86 showed the opposite trend. Data were represented as mean ± SEM. ns, not signifcant; *, p<0.05; **, p<0.01; ***, p<0.001.

GDF15 has been implicated in shaping immunosuppressive tumor microenvironments (TME) and promoting radioresistance in various cancers 36, 51-53, but its role in GBM remained unclear. Analysis of publicly available databases revealed that high GDF15 expression correlated with an increased M2/M1 macrophage ratio, higher Treg infiltration, and elevated neutrophil infiltration (Supplementary Fig.5A). To validate these findings *in vivo*, we first generated GDF15-overexpressing murine glioma GL261 cells (Fig. 5A), and a colony formation assay confirmed their enhanced resistance to radiation (Fig.5B) *in vitro*. These cells were then implanted into the brains of C57BL/6 mice, which subsequently received radiation therapy. Tumor tissues from GDF15-overexpressing immunocompetent mice after irradiation were collected for further analysis. Immunohistochemistry and flow cytometry confirmed a significantly increase in M2 macrophage infiltration in the GDF15 overexpressing group (Fig. 5C-E). To confirm translational relevance in human GBM, transcriptomic analysis on LN229 cells with sh-GDF15 under radiation conditions revealed enrichment of pathways associated with M2 macrophage polarization (Fig. 6A). Public dataset analysis further showed a significantly positive correlation between GDF15 and C206 expression in human GBM tissues (Fig. 6B). In the nude mouse xenograft model of human GBM, *in vivo* validation further demonstrated that GDF15 overexpression increased M2 macrophage infiltration, whereas GDF15 knockdown reduced M2 macrophage accumulation in human GBM tissues (Fig. 6C-D, Supplementary Fig. 3E, I, J and K, O, P). *In vitro*, co-culture of macrophages with tumor cell supernatants (Fig. 6E) demonstrated upregulation of M2 polarization markers by qRT-PCR (Fig. 6F) and increased M2/M1 ratio by flow cytometry (Fig. 6G), confirming that GDF15 promotes M2 macrophage polarization and infiltration in GBM.

## Discussion

Radiotherapy remains a cornerstone in GBM treatment, but the development of radioresistance markedly limits its efficacy, resulting in tumor recurrence and poor prognosis 1-3. In this study, we identified GDF15, a stress-inducible cytokine, as a key mediator of radioresistance in GBM. Previous studies have demonstrated that GDF15 is rapidly upregulated following radiotherapy and other cellular stresses in multiple tumor types, where it functions as an adaptive survival factor supporting treatment resistance 54-56. Consistently, our experimental results show that GDF15 expression in GBM cells is upregulated in a time- and dose-dependent manner following radiation (Supplementary Fig. 1G-H). Moreover, we found that GDF15 expression was elevated in radioresistant GBM cell lines and recurrent GBM tissues, a pattern consistently observed in transcriptomic data from NCBI GEO (Fig. 1B-F). Functional experiments demonstrated that GDF15 overexpression enhanced GBM cell resistance to irradiation both *in vitro* and *in vivo*, while GDF15 knockdown sensitized cells to radiation. These results established GDF15 as a pivotal driver of radioresistance in GBM.

Mechanistically, radiotherapy triggers diverse cell death pathways, including apoptosis, necrosis, and autophagy 57-59. Ferroptosis has been considered a key determinant of radiotherapy resistance, and GDF15 has been implicated as a ferroptosis inhibitor; however, its role in GBM radioresistance was previously unexplored 15, 60. In our study, GDF15 overexpression enhanced cellular GSH levels and reduced lipid peroxidation post-radiation, as indicated by lower MDA levels and decreased ferroptotic features in transmission electron microscopy. Conversely, GDF15 knockdown increased lipid peroxidation and induced ferroptosis, suggesting that GDF15 inhibits ferroptosis to promote GBM radioresistance. Among the regulators of ferroptosis, NRF2 is a pivotal transcription factor controlling antioxidant genes, including SLC7A11 and GPX4, to protect cells from iron-dependent lipid peroxidation 45-48, 50. Prior studies have identified the GDF15-NRF2 axis as a potential therapeutic target in treatment-resistant cancers 17, 61-63. We therefore examined the relationship between GDF15 and NRF2 in GBM. GDF15 did not affect NRF2 mRNA levels but increased NRF2 protein expression, indicating post-transcriptional regulation. Mechanistically, GDF15 stabilized NRF2 by inhibiting its ubiquitination, thus promoting NRF2-mediated protection against ferroptosis. These findings suggest that GDF15 acts as an NRF2 stabilizer, suppressing ferroptosis and enhancing GBM radioresistance. In addition to direct tumor-intrinsic mechanisms, radioresistance is profoundly influenced by the tumor microenvironment (TME). GDF15 has been reported to promote immunosuppressive remodeling in various cancers by modulating immune cell function, including macrophage polarization and T cell responses 28, 30, 31, 51. In our *in vivo* studies, GDF15 overexpression post-radiation promoted M2 macrophage polarization in the GBM microenvironment, shifting the TME toward an immunosuppressive phenotype. These findings suggest that GDF15 may contribute to immune evasion, thereby enhancing GBM radioresistance.

Targeting GDF15 has emerged as a promising therapeutic strategy to improve radiation response in GBM through the induction of ferroptosis and reversal of immunosuppressive mechanisms-such as reduced M2 macrophage polarization. Preclinical studies have demonstrated the feasibility of this approach using neutralizing antibodies, RNA-based therapies, or advanced delivery systems, including nanoparticles for CRISPR/Cas9-mediated gene knockout and siRNA-loaded exosomes 52, 64-66. These systems have shown efficient blood-brain barrier penetration, robust tumor growth suppression, and synergistic effects with anti-PD-1 therapy. However, key challenges, including optimal treatment sequencing, dosing regimens, and patient stratification based on GDF15 expression levels, require further investigation before clinical translation. Although mechanistically compelling, the combination of GDF15 inhibition and radiotherapy necessitates additional validation in physiologically relevant models and early-phase clinical trials.

This study has several limitations. First, although we demonstrated that GDF15 stabilizes NRF2 by inhibiting its ubiquitination, the precise molecular intermediates, such as specific E3 ligases or cofactors, remain unknown. Second, although we demonstrated that GDF15 promotes GBM radioresistance through M2 macrophage infiltration and polarization, the detailed molecular mechanisms remain unclear. In particular, whether this immunosuppressive remodeling is linked to GDF15-mediated inhibition of ferroptosis warrants further investigation. Finally, our findings are primarily based on preclinical models; clinical validation will be essential to confirm the translational relevance of targeting GDF15 in GBM radiotherapy.

In summary, our study identifies GDF15 as a critical mediator of radioresistance in GBM. GDF15 promotes radioresistance through multiple mechanisms, including inhibition of ferroptosis via NRF2 stabilization and modulation of the tumor immune microenvironment. Targeting GDF15 may provide a novel therapeutic strategy to improve GBM treatment outcomes.

## Supplementary Material

Supplementary figures and table.
