# Supplementary material for: GDF15 Drives Glioblastoma Radioresistance by Inhibiting Ferroptosis and Remodeling the Immune Microenvironment
Source: Int J Biol Sci. 2025 Oct 20;21(15):6794–807. doi: 10.7150/ijbs.115721 (PMC12631175; doi:10.7150/ijbs.115721)
Supplement: Supplementary file 1 — Supplementary figures and table. [file ijbsv21p6794s1.pdf]

Supplementary Material

GDF15 Drives Glioblastoma Radioresistance by Inhibiting Ferroptosis and Remodeling the Immune Microenvironment

Wenqing Feng[1†], Yantan Liu[1†], Qinghua Zhang[1†], Shushu Hu[1], Dehuang Xie[1], Peixin Tan[1], Yuan Lei[1], Chen Chen[1], Chen Ren[1]*, Shasha Du[1]*

1. Department of Radiation Oncology, Guangdong Provincial People's Hospital (Guangdong Academy of Medical Sciences), Southern Medical University, Guangzhou, 510080, Guangdong, China

†These authors contributed equally to this study.

*Corresponding authors:

Chen Ren, MD, PhD, Department of Radiation Oncology, Guangdong Provincial People's Hospital, Guangdong Academy of Medical Sciences, Guangzhou, Guangdong 510080, P.R. China;

E-mail: renchen@smu.edu.cn

Sha-sha Du, MD, PhD, Department of Radiation Oncology, Guangdong Provincial People's Hospital, Guangdong Academy of Medical Sciences, Guangzhou, Guangdong 510080, P.R. China;

E-mail: dushasha8557@smu.edu.cn

Supplementary Tables

Supplementary Table 1

Comprehensive details of the primary and secondary antibodies used in this study for Western blot (WB), immunofluorescence (IF), flow cytometry (FC), and immunohistochemistry (IHC) assays are provided.

Supplementary Table1: Antibody List for WB/IF/IHC/FCM

Name	Application	Dilution	Catalog Number/Ventor
γ-H2AX	IF/WB	IF：1:2000<br>WB：1:1000	AP0687/ABclonal
Goat Anti-Rabbit IgG H&L (Alexa Fluor® 488)	IF	IF：1:1000	ab150077/Abcam
NRF2	WB	WB:1:1000	HA721432/HUABIO
NRF2	WB/IP	IP: 2ug	30000-0-AP/Proteintech

GDF15	WB	WB: 1:500<br>IP: 2ug	sc-377195/Santa Cruz
GDF15	WB	WB:1000	ER1909-46/HUABIO
GDF15	IHC	IHC：1:400	ER1909-46/HUABIO
Tubulin	WB	WB: 1:1000	FD0064-50/Fudebio
Tubulin	WB	WB: 1:1000	bsm-33039M/Bioss
Flag	WB	WB1:1000	AE063/ABclonal
Myc	WB	WB1:1000	AE070/ABclonal
UB	WB	WB1:1000	ET1609-21/HUABIO
GPX4	WB	WB1:1000	ab125066/Abcam
Goat Anti-Rabbit IgG H&L (HRP)	WB	WB1:5000	ab6721/Abcam
Goat Anti-Mouse IgG H&L (HRP)	WB	WB1:5000	ab6789/Abcam
Ki67	IHC	IHC：1:15000	ET1609-34/HUABIO
4-HNE	IHC	IHC：1:400	bs-6313R/Bioss
SLC7A11	IHC	IHC:1:400	A13685/ABclonal
CD8	IHC	IHC:1:400	#19589/CST
CD86	IHC	IHC:1:400	#19589/CST
CD86	IHC	IHC:1:100	91882S/Abcam
CD206	IHC	IHC:1:400	#24595/CST
FOXP3	IHC	IHC:1:400	HA722835/HUABIO
MPO	IHC	IHC 1:500	Ab208670/Abcam
IgG- Rabbit	IP	IP：2ug	A7016/Beyotime

IgG- Mouse	IP	IP：2ug	A7028/Beyotime
CD11b	FCM	1:500	557397/BD Pharmingen
CD45	FCM	1:500	103107/Biolegend
anti-mouse CD16/32	FCM	1:500	156603/Biolegend
Anti-Mouse F4/80	FCM	1:1000	565411/BD Pharmingen
anti-mouse CD206	FCM	1:500	141708/Biolegend
anti-mouse CD86	FCM	1:500	560582/BD Pharmingen
Zombie NIR™ Fixable Viability Kit	FCM	1:1000	423105/Biolegend

Supplementary Table 2

The primer sequences utilized for quantitative real-time polymerase chain reaction (qPCR) analysis in this study.

Supplementary Table 2. Primers for qPCR

Gene	Forward	Reverse
INOS1	CGCATGACCTTGGTGTTTGG	CATAGACCTTGGGCTTGCCA
IL1B	CCAGCTACGAATCTCCG	CGTTATCCCATGTGTCG
IL6	CTTCGGTCCAGTTGCCTTCT	GGTGAGTGGCTGTCTGTGTG
ARG	CTTGGCAAAAGACTTATCCTTAG	ATGACATGGACACATAGTACCTTTC
CD86	CCCCAGACCACATTCCTTGG	TGTTCACTCTCTTCCCTCTCCA
CD206	TACTGAACCCCCACAACTGC	ACCAGAGAGGAACCCATTCG
CD163	GCTCAGGAAACCAGTCCCAA	TACCAGGCGAAGTTGACCAC
NRF2	TCAGCGACGGAAAGAGTATGA	CCACTGGTTTCTGACTGGATGT
GDF15	GACCCTCAGAGTTGCACTCC	GCCTGGTTAGCAGGTCCTC
GAPDH	GGAGCGAGATCCCTCCAAAAT	GGCTGTTGTCATACTTCTCATGG

Supplementary Figure Legends

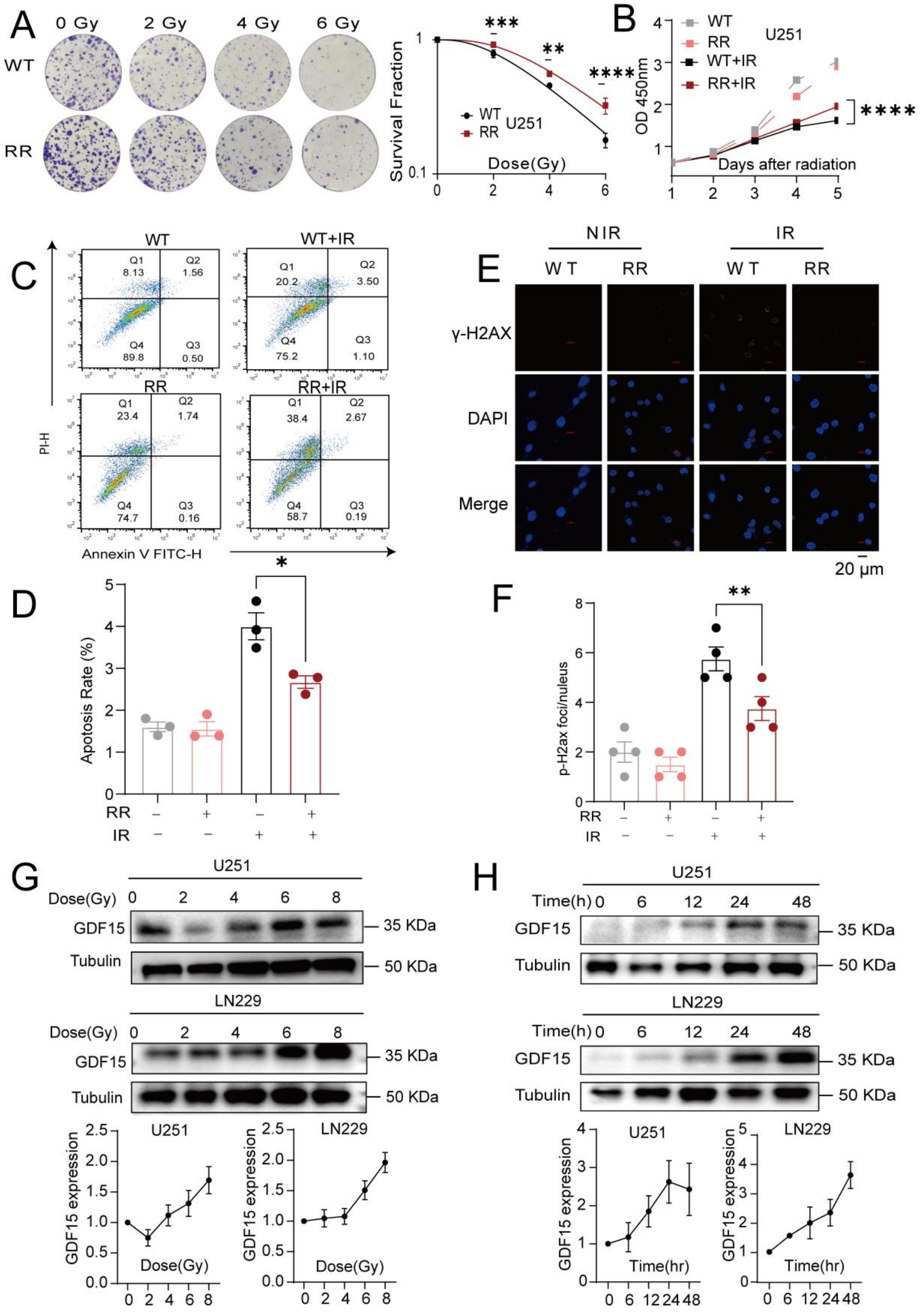

Supplementary Fig. 1.| Validation of radioresistant GBM models and dynamic GDF15 expression under radiation.

(A) Representative colony formation assays confirming the successful establishment of radioresistant U251 cells. Surviving colonies were quantified and plotted. Three independent experiments performed

in triplicate.

(B) CCK-8 assay confirming the establishment of radioresistant U251 cells.

(C-D) Flow cytometry analysis of apoptosis in radioresistant U251 cells with or without iradiation.IR=8 Gy.

(E-F) Immunofluorescence analysis was conducted to quantify γ-H2AX foci formation in radioresistant U251 cells (scale bar = 20 μm).

(G-H) Western blots showing dose-dependent changes in GDF15 expression following gradient irradiation (0, 2, 4, 6, 8 Gy) and time-course changes after a single 4 Gy irradiation in LN229 and U251 cells. Data represent mean ± SEM of three independent experiments. ns, not significant; *p < 0.05; **p < 0.01; ***p < 0.001.

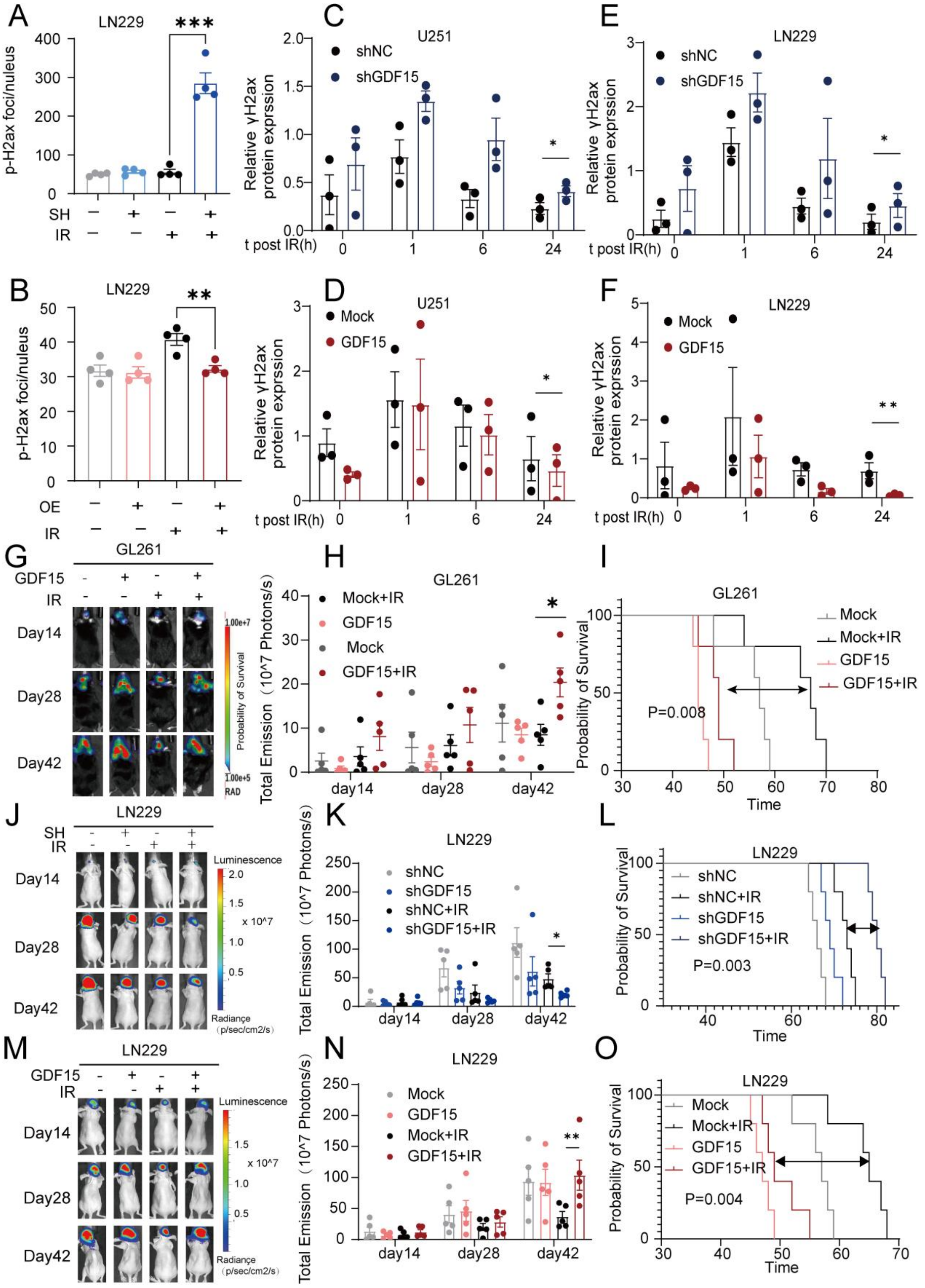

Supplementary Fig. 2.| GDF15 contributes to radiation resistance in GBM in vitro and in vivo.

(A-B) Immunofluorescence analysis was conducted to quantify γ-H2AX foci formation in LN229 cells following GDF15 modulation under 8 Gy X-ray irradiation at 24 hours. (scale bar = 20 μm). Quantitative analysis illustrates the average number of foci per cell across 4 randomly selected images under each condition.

(C-F) Quantification of γ-H2AX protein levels from Western blotting in U251 and LN229 cells with GDF15 knockdown or overexpression after irradiation(8 Gy). Data are mean ± SEM of three independent experiments.

(G) Representative in vivo bioluminescence imaging (BLI) of GL261 tumors overexpressing GDF15 at weeks 2, 4, and 6 post-implantation. Each group included 5 mice (n = 5 per group).

(H) Quantification of BLI signal intensity corresponding to panel G, highlighting differential tumor growth rates among groups. Each group included 5 mice (n = 5 per group).

(I) Kaplan–Meier survival curves of mice implanted with GL261 cells under the indicated treatments. Each group included 5 mice (n = 5 per group). Survival differences were assessed by log-rank test.

(J,M) Representative in vivo BLI at weeks 2, 4, and 6 post-implantation in nude mice bearing LN229 tumors with GDF15 knockdown or overexpression, with or without 10 Gy irradiation. Each group included 5 mice (n = 5).

(K,N) Quantification of BLI signal intensity corresponding to panels J and M, highlighting differential tumor growth rates among groups. Each group included 5 mice (n = 5 per group).

(L,O) Kaplan-Meier survival curves of nude mice implanted with LN229 cells under the indicated treatments. Each group included 5 mice (n = 5 per group). Survival differences were assessed by log-rank test. Data are mean ± SEM. ns, not significant; *p < 0.05; **p < 0.01; ***p < 0.001.

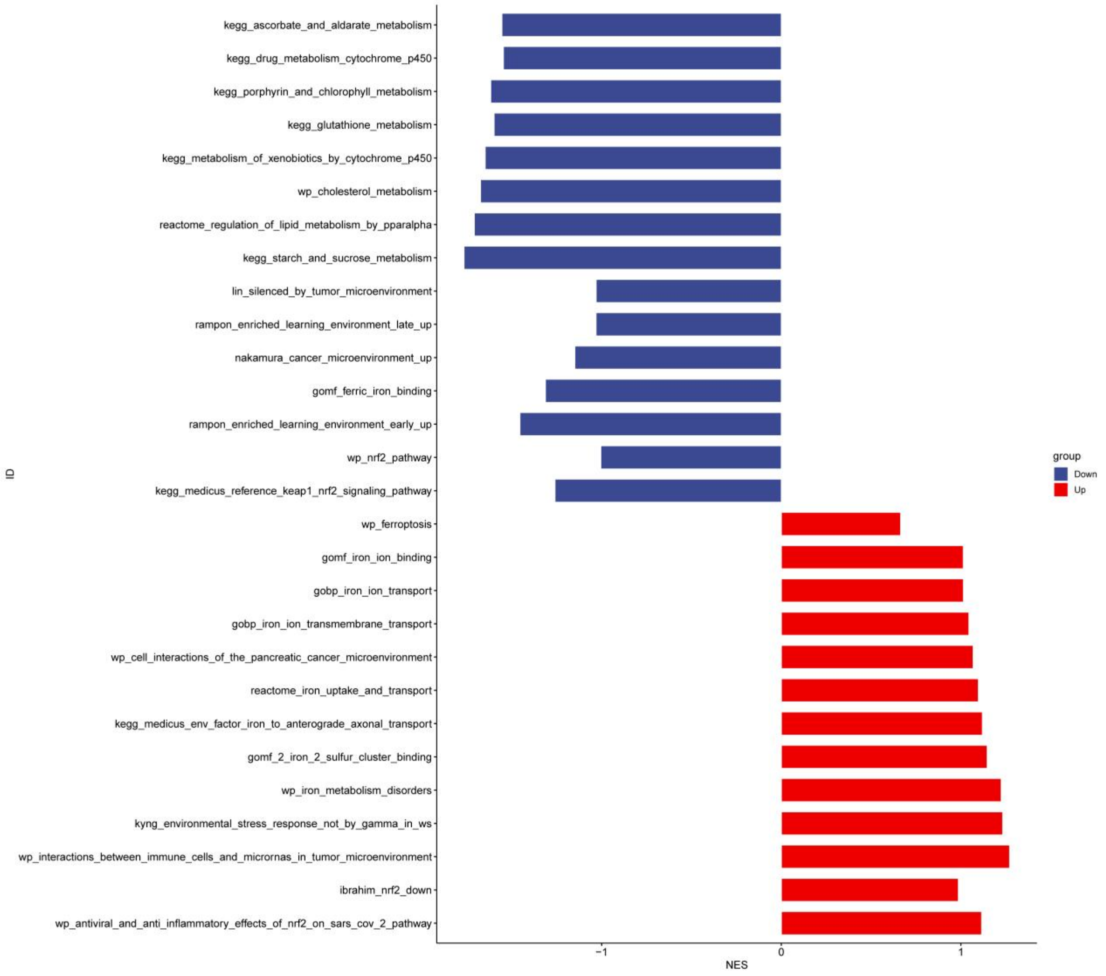

Supplementary Fig3.1| Pathway enrichment in shGDF15 LN229 cells

Bar plot of pathway enrichment analysis for differentially expressed genes in LN229 cells with GDF15 knockdown (shGDF15). Red bars denote upregulated pathways, while blue bars denote downregulated

pathways.

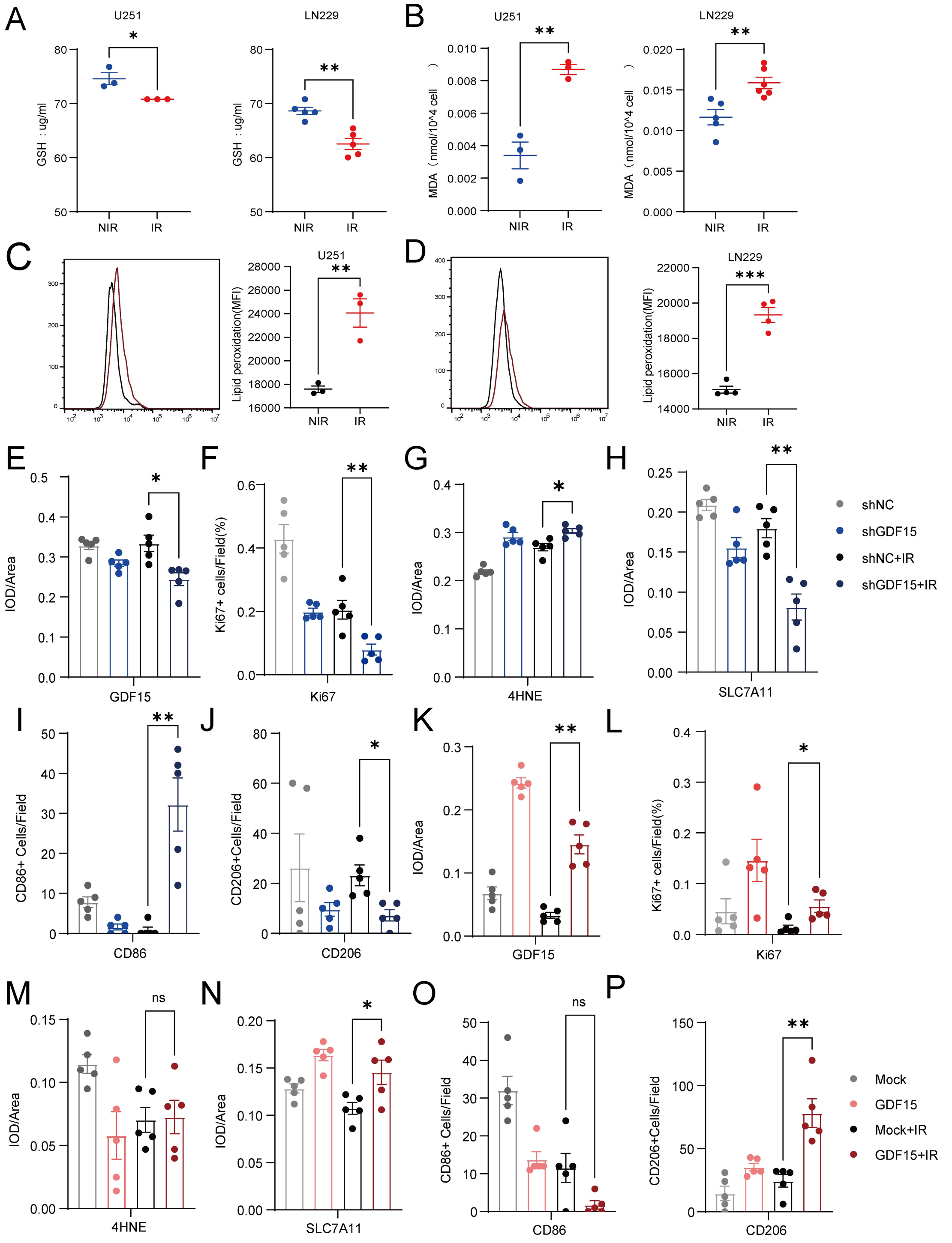

Supplementary Fig3. | GDF15 inhibits radiotherapy-induced ferroptosis in GBM cells

(A)Detection of GSH levels after 8 Gy irradiation in U251/LN229 cells. IR=8 Gy.

(B)Detection of MDA levels after 8 Gy irradiation in U251/LN229 cells. IR=8 Gy.

(C-D) Detection of lipid peroxidation levels after 8 Gy irradiation in U251/LN229 cells.

(E-P) Quantification of IHC staining for GDF15, Ki67, 4-HNE, SLC7A11, CD86, and CD206 in U251 xenograft tumors under indicated treatments. These quantitative data correspond to Figure 3 F-I and

Figure 6 C-D. Data were represented as mean ± SEM. ns, not signifcant; *, p<0.05; **, p<0.01; ***, p<0.001

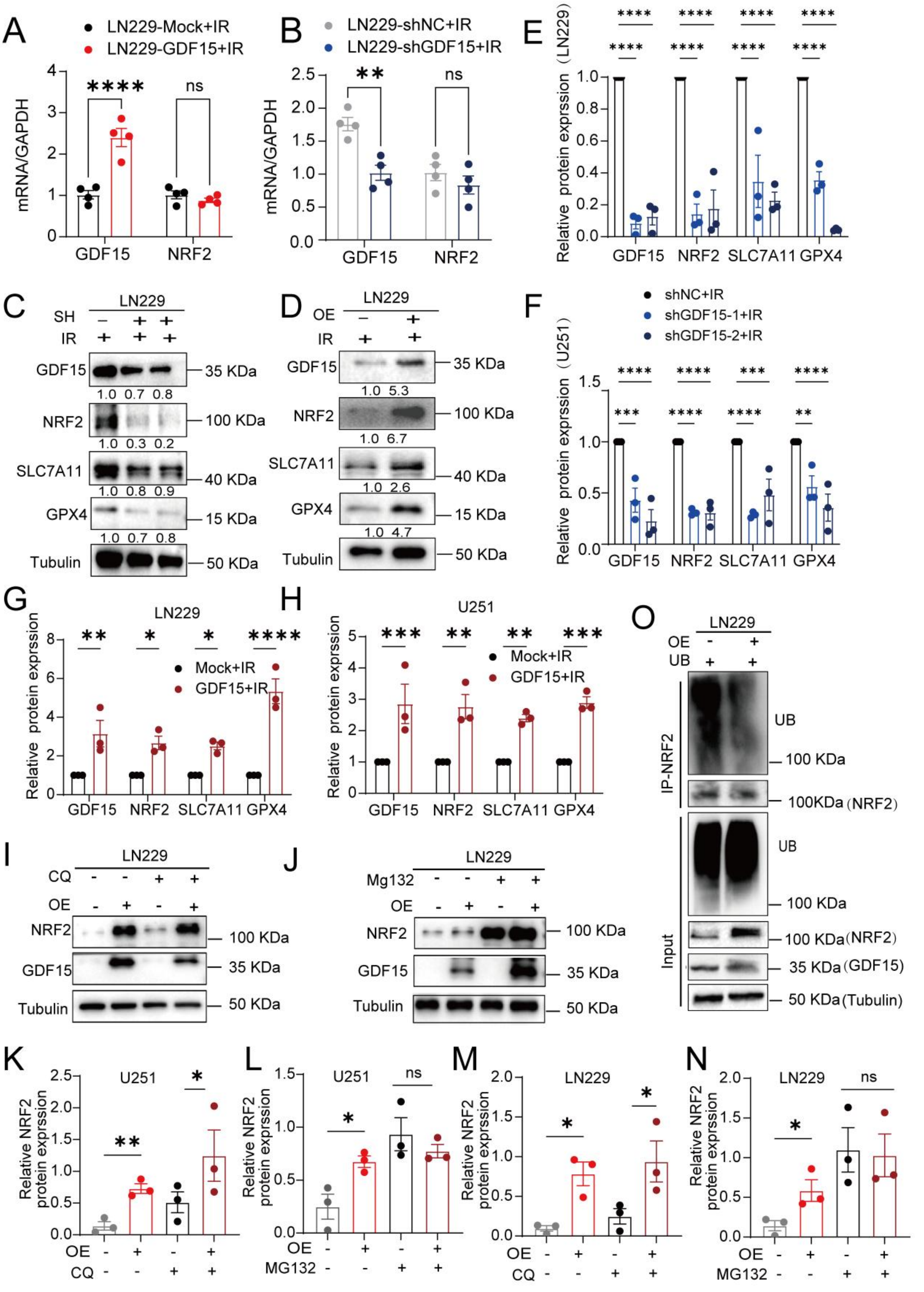

Supplementary Fig4. | GDF15 Suppresses Ferroptosis in GBM via the NRF2/SLC7A11/GPX4 Pathway.

(A-B) qRT–PCR analysis of the mRNA expression levels of NRF2 from GDF15 knockdown or overexpression in LN229 cells following radiation. IR=8 Gy.

(C-H) Western blot analysis of the protein expression levels of NRF2, SLC7A11, GPX4 from GDF15 knockdown or overexpression in LN229 and U251 cells following radiation. IR=8 Gy.

(I-J) NRF2 detection in LN299 cells transfected with GDF15 overexpression; each group was treated with or without proteasome inhibitor MG132 or lysosomal inhibitor CQ.

(K-N) Quantification of WB of NRF2 detection in U251 or LN229 cells transfected with GDF15 overexpression; each group was treated with or without proteasome inhibitor MG132 or lysosomal inhibitor CQ.

(O) GDF15 was stably overexpressed in LN229 cells. NRF2-IP antibody was used to immunoprecipitate endogenous NRF2 protein, and ubiquitinated NRF2 in the immunocomplexes was detected with Ub antibody via WB assay. Data were represented as mean ± SEM. ns, not signifcant; *, p<0.05; **, p<0.01; ***, p<0.001

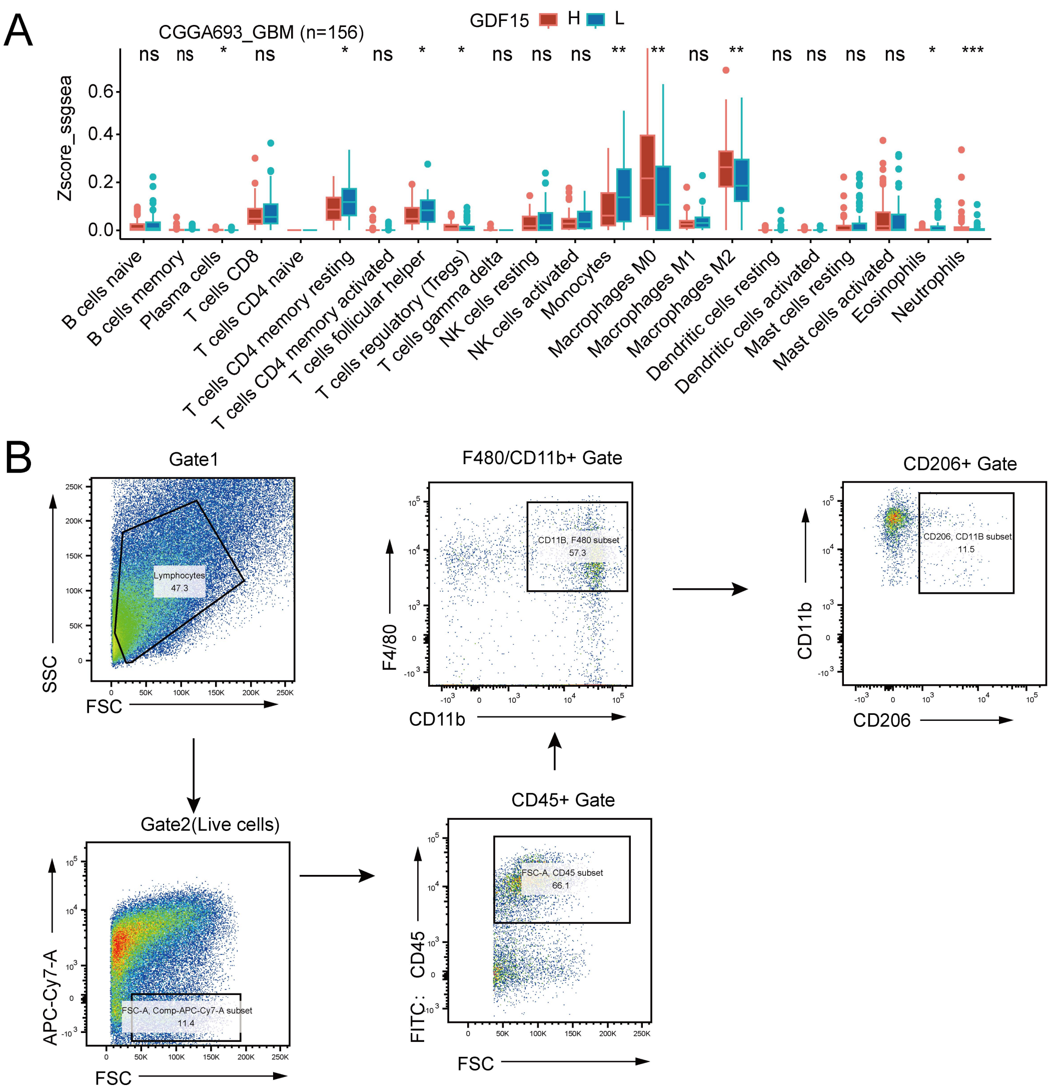

Supplementary Fig5. | GDF15 mediates radiotherapy resistance by promoting M2 macrophage-driven immunosuppression.

(A) CIBERSORT analysis of immune cell infiltration in GBM tissues from the CGGA693 cohort (GBM=156) stratified by GDF15 expression (High vs Low, defined by median cutoff, two-tailed

t-test).

(B) Flow cytometry gating strategy of Figure 5 E.

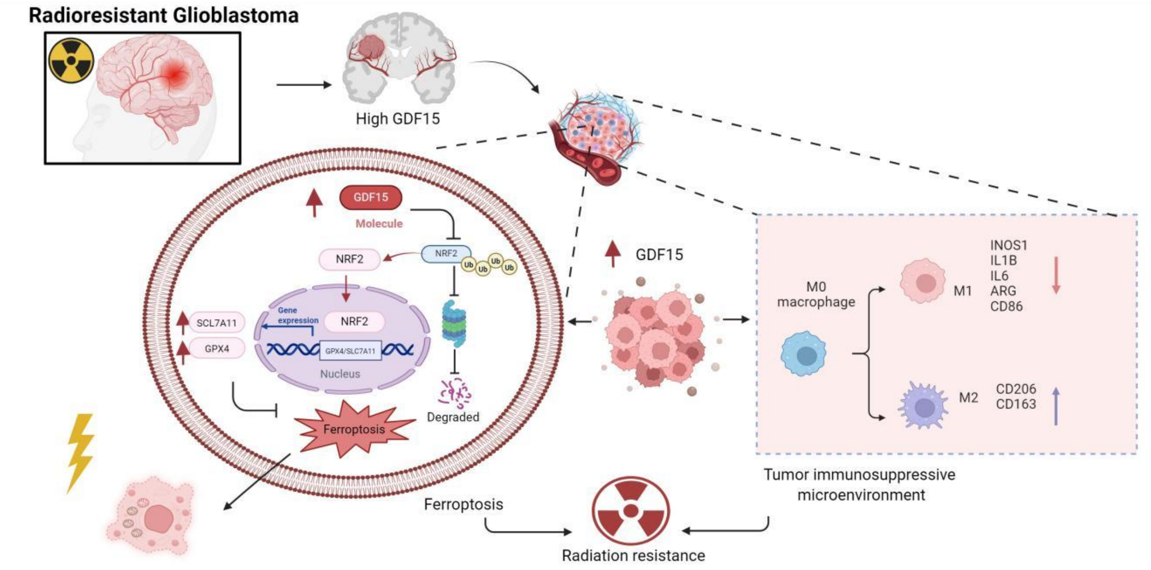

Supplementary Fig6. | Working model of GDF15-mediated radioresistance in Glioblastoma.

GDF15 promotes glioblastoma radioresistance through dual mechanisms: suppressing radiation-induced ferroptosis via NRF2 stabilization to block lipid peroxidation and reprogramming the immunosuppressive tumor microenvironment by recruiting M2 macrophages. Created with BioRender.com.
